# Naphthyridine carbamate dimer ligand induces formation of Z-RNA-like fold of disease-related RNA and exhibits a molecular glue characteristics in crystal lattice formation

**DOI:** 10.1093/nar/gkaf924

**Published:** 2025-09-17

**Authors:** Martyna Mateja-Pluta, Leszek Błaszczyk, Magdalena Bejger, Kazuhiko Nakatani, Agnieszka Kiliszek

**Affiliations:** Institute of Bioorganic Chemistry, Polish Academy of Sciences, Z. Noskowskiego 12/14, 61-704, Poland; Institute of Bioorganic Chemistry, Polish Academy of Sciences, Z. Noskowskiego 12/14, 61-704, Poland; Institute of Bioorganic Chemistry, Polish Academy of Sciences, Z. Noskowskiego 12/14, 61-704, Poland; Department of Regulatory Bioorganic Chemistry, SANKEN (The Institute of Scientific and Industrial Research), Osaka University, 8-1 Mihogaoka, Ibaraki 567-0047, Japan; Institute of Bioorganic Chemistry, Polish Academy of Sciences, Z. Noskowskiego 12/14, 61-704, Poland

## Abstract

The naphthyridine carbamate dimer (NCD) is a small molecule that recognizes disease-related RNA containing UGGAA repeats associated with spinocerebellar ataxia type 31 (SCA 31) and alleviates the disease phenotype *in vitro* and *in vivo*. In this study, we use X-ray crystallography to elucidate the mode of NCD binding in detail. We determine the crystal structures of the RNA–NCD complex and a structure of unliganded RNA. The NCD interacts differently than in previously reported nuclear magnetic resonance structure, forming pseudo-canonical base pairs with guanosine residues located on the same RNA strand. Furthermore, in one of the complexes, the ligand is located between symmetry-related RNA molecules, exhibiting a molecular glue characteristics in crystal lattice formation. The comparison of RNA–NCD and ligand-free models allows the identification of structural changes in RNA upon ligand binding from A-form to Z-RNA-like form. These observations extend our understanding of the interactions between RNA and small compounds and can be useful as a reference model in the development of bioinformatics tools for RNA–ligand structure predictions.

## Graphical Abstract

**Graphical Abstract:**
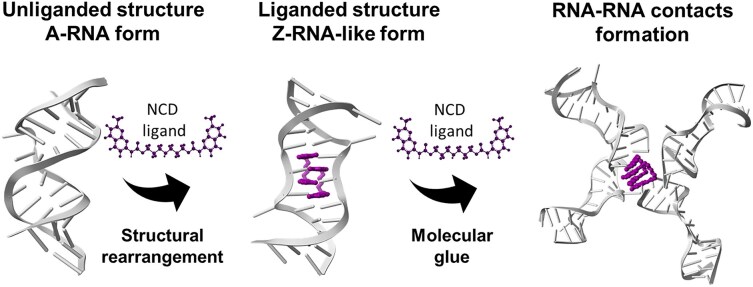

## Introduction

Biologically active small synthetic molecules are organic compounds with molecular sizes typically <1000 Da [1]. Since the synthesis of urea in 1828, the development of synthetic molecules has evolved into a sophisticated research field supported by structural methods, such as X-ray crystallography, nuclear magnetic resonance (NMR), and bioinformatics. Currently, it is experiencing a breakthrough, mainly due to innovations in new moieties, implementation of high-throughput techniques, and advances in bioinformatics [2–6].

New modalities have provided an opportunity to focus on the underexplored field of RNA-targeting. RNA is a vital molecule that is abundant in cells and is involved in almost all steps of gene expression. Alterations in the RNA structure and function can lead to the development of serious disorders. In this regard, RNA is an attractive target for drug development and an exciting area of many ongoing studies. However, there are significant challenges and risks, including target selection, hit validation, optimization of small molecules, and translation into the cellular environment. One of the difficulties arises from the specific structural characteristics of the RNA, which is a negatively charged molecule that prefers to bind ligands with positively charged functional groups or metal ions, or water molecules from the environment. Another aspect concerns the architecture of the binding pockets in RNA, which are different from that observed in proteins. Typically, they are shallow, polar and solvated [7]. Moreover, RNA exhibits conformational dynamics that can lead to the coexistence of different structures of the same molecule, or structural rearrangements upon ligand binding [8]. Finally, our understanding of the structural nature of RNA–ligand interactions is very limited. There are >37 000 biomolecule structures in complex with ligands deposited in the Protein Data Bank, but only 565 represent RNA–ligand complexes. Although experimentally determined structures provide invaluable knowledge that supports the selection of compounds from libraries for high-throughput screening, optimization of small molecules and the improvement of bioinformatics structural predictions could also accelerate this process.

In recent years, we developed a class of small molecules called mismatch binding ligands (MBLs) that recognize noncanonical base pairs in RNA or mismatches in DNA [9–13]. They were designed to contain aromatic moieties complementary to the hydrogen-bonding pattern of the nucleobases [11,14]. Structural analysis using NMR and X-ray crystallography confirmed that MBLs interact directly with nucleotides forming Watson–Crick like pairs [8, 11, 12, 15, 16]. In some cases, ligand binding was associated with conformational changes in the nucleic acid structure [11, 12]. Recently, one of the first-generation molecules, naphthyridine carbamate dimer (NCD), was shown to recognize the pathogenic RNA associated with spinocerebellar ataxia type 31 (SCA 31) [17]. SCA 31 is an autosomal spinocerebellar neurodegenerative disorder. It is caused by abnormally repeated 5′-UGGAA-3′/5′-UGGAA-3′ RNA pentads, which sequester many cellular proteins by forming toxic nuclear foci [18]. *In vitro and in vivo* studies have shown that NCD ligand possesses pharmaceutical properties. Feeding NCD to larvae of the Drosophila SCA31 model alleviated the disease phenotype induced by toxic UGGAA repeats. The previous studies have demonstrated that NCD acts as a molecular glue facilitating the assembly and stabilization of higher-order RNA and DNA architectures. NCD and its multimeric derivatives were used to induce folding of DNA into tetrahedron structure, for modulation of RNA aptamer activity and to enhance the catalytic properties of the hammerhead ribozyme [19–22]. An NMR study showed that two NCD molecules bind to 5′-UGGAA-3′/5′-UGGAA-3′ RNA. Each NCD ligand interacted with two guanosine residues located on opposite RNA strands and formed noncanonical guanosine–naphthyridine pairs [17]. To further elucidate the mode of NCD binding, we performed an X-ray crystallographic analysis of RNA oligomers containing the 5′-UGGAA-3′/5′-UGGAA-3′ motif.

Here, we report two crystal structures of the RNA–NCD complex and one, so far unreported, structure of unliganded RNA. In the models of complexes, we observed two NCD molecules bound to the internal loop of the UGGAA motif. The mode of binding is different from that of previously reported NMR structure. Furthermore, in one of the complexes, an additional binding site for NCD was found. The ligand was located between the symmetry-related RNA molecules and helped forming additional interactions in the crystal lattice. This shows that NCD exhibits a molecular glue characteristics and facilitate the formation of crystal contacts. In the unliganded model, the 5′-UGGAA-3′/5′-UGGAA-3′ motif formed an internal loop that maintained the helical character of RNA. Three out of the four guanosine residues of the motif stacked one above another, attracting the cobaltamine [Co(NH_3_)_6_]^3+^ cation. The comparison between bound and unbound models revealed that the binding of the NCD molecules caused major structural changes in the RNA which were confirmed by biophysical methods. These observations extend our understanding of the interactions between RNA and small compounds in the context of potential therapeutics against RNA-driven disorders, and can be useful as a reference model in the development of bioinformatics tools for RNA–ligand structure predictions.

## Materials and methods

### Synthesis, purification, and crystallization of the oligomers

All oligomers were synthesized by the solid-phase method in an Applied Biosystem DNA/RNA synthesizer using TOM-protected phosphoramidites [23]. The synthesis was conducted using the DMT-ON procedure, leading to oligomers with a trityl group attached at the 5′end. The RNA oligonucleotides were purified using Glen-Pak cartridges according to the appropriate protocol, desalted, and lyophilized under vacuum conditions using Speed-Vac [24–26]. Prior to crystallization, RNA was dissolved in 10 mM sodium cacodylate (pH 7.0) to a final concentration of 1 mM. Oligonucleotides were denatured at 95°C for 5 min and then gradually cooled to room temperature. Crystallization of RNA complexes with NCDs was conducted using the sitting drop vapor-diffusion technique at 19°C. RNA and NCD ligands were mixed in a 1:2 molar ratio. The optimal crystallization conditions for ROG2 oligomer were 0.012 M sodium chloride, 0.08 M potassium chloride, 0.04 M sodium cacodylate trihydrate (pH 5.5), 45% v/v (±)-2-methyl-2,4-pentanediol, 0.002 M hexammine cobalt(III) chloride, for ROG2–NCD 0.08 M potassium chloride, 0.02 M magnesium chloride hexahydrate, 0.04 M sodium cacodylate trihydrate (pH 6.0), 45% v/v (±)-2-methyl-2,4-pentanediol, 0.012 M spermine tetrahydrochloride and for ROG1–NCD 0.01 M magnesium acetate tetrahydrate, 0.05 M sodium cacodylate trihydrate (pH 6.5), 1.3 M lithium sulfate monohydrate. Crystals of ROG1–NCD appeared within 3 months, ROG2–NCD within 1 month while unliganded ROG2 within 2 weeks. The crystals of ROG1–NCD were cryoprotected with 20% glycerol and flash frozen, whereas ROG2–NCD and ROG2 were directly transferred from the crystallization drop to liquid nitrogen. The crystals were transferred to a synchrotron core facility by using a dry shipper.

### X-ray data collection, structure solution, and refinement

The X-ray diffraction data were obtained using the beam lines at BESSY II (Berlin) [27–29], DESY (Hamburg) [30, 31], and ESRF (Grenoble) [32, 33]. The data were integrated and scaled using the XDS program suite, facilitated by the xdsgui interface [34]. The complex structure of ROG1–NCD complex was solved by the molecular replacement (MR) method using the PHASER program [35, 36]. The search model was the crystal structure of the CUG repeat duplex (PDB code: 3GLP) [37]. The structures of ROG2–NCD complex and ROG2 oligomer were solved by MR with PHASER, using ROG1–NCD (PDB code: 9I9W) as a search model. The refinement were carried out using Refmac5 from the CCP4 program suite [38–42]. Restraints for the NCD ligand were generated using the Grade Web server, a tool developed by Global Phasing (https://grade.globalphasing.org). Manual model building and electron density map inspection were conducted using Coot [43, 44]. The helical parameters were calculated using 3DNA [45, 46]. The X-ray data and refinement statistics are summarized in Supplementary Table S1.

### Circular dichroism measurement

Circular dichroism (CD) experiments were carried out on a J-725 CD spectropolarimeter (JASCO) using a 10 mm path length cell at room temperature. Prior to measurement, RNA (10 μM) was refolded in sodium cacodylate buffer (40 mM, pH 7.0) containing 100 mM NaCl for 5 min at 95°C and snap-cooled on ice for 10 min, followed by incubation for 10 min at room temperature. The CD titration spectra of ROG2 with variable concentrations of NCD (10, 20, 30, 40, and 50 μM) were measured at ambient temperature. For each sample, five spectral scans were accumulated in the 215–370 nm range.

### Differential scanning calorimetry measurements

The RNA oligomers were dissolved in 100 mM sodium chloride and 40 mM sodium cacodylate buffer adjusted to pH 7.0. The final concentration of RNA was 100 μM with presence and absence of NCD ligand (200 μM) and cobaltamine chloride (200 μM) To equilibrate the RNA with the reference solutions, the samples were dialyzed overnight at 4°C. Differential scanning calorimetry (DSC) experiments were conducted using a MicroCal PEAQ-DSC calorimeter (Malvern Instruments, Ltd.). Each measurement was carried out in five cycles of heating and cooling in the range of 2–110°C at a scan rate of 1°C/min. Initially, reference scans of the buffer were performed to establish the instrument’s thermal history and achieve near-perfect baseline repeatability. The results were analyzed using dedicated software implemented by Malvern Instruments. The melting temperature (*T*_m_) was calculated by applying two-state model fitting.

### Ultraviolet melting temperature measurements

Ultraviolet (UV) thermal melting studies were performed on a Jasco V-700 spectrometer with a thermoprogrammer. The RNA oligomers were dissolved in a buffer containing 100 mM sodium chloride and 40 mM sodium cacodylate (pH 7.0). Unliganded duplexes of ROG1 and ROG2 oligomer as well as RNA–Co(NH₃)₆ and RNA–NCD complexes were prepared in three different concentrations (10, 30, and 100  μM). The ratio between RNA and ligand molecules (NCD and cobaltammine) was 1:2. The UV absorption versus temperature was measured at 260 nm at a heating rate of 0.5°C/min in the range 4–90°C [47]. The melting curves were analyzed using Microsoft Excel software to calculate the first derivative of the UV-melting curves and melting temperature.

### Native and denaturing gel electrophoresis

For native gel electrophoresis 10 pmol of RNA in 10 mM sodium cacodylate (pH 7.0) and 100 mM sodium chloride was renatured for 2 min at 95°C, snap cooled in 4°C for 5 min and transferred into room temperature. Next, RNA was mixed with increasing concentrations of NCD ligand (0.62–80  μM) and incubated for 12 h at room temperature. The final RNA concentration in the reaction was 0.3  μM. Samples were combined with ficoll (2%) and loaded on 20% polyacrylamide gel with 0.5× TB buffer (Tris-Borate). After electrophoresis gel was stained with SYBR Gold and visualized using Amersham Typhoon Biomolecular Imager (Cytiva). For evaluation of RNA homogeneity samples were renatured in the same manner except that 10, 30, 100 μM RNA concentration was used. RNA was visualized using toluidine blue staining.

In case of denaturing gel electrophoresis 1 nmol of RNA was combined with equal volume of 8 M urea and loaded on 15% polyacrylamide gel in 1× TBE buffer (Tris-Borate-EDTA). After electrophoresis RNA was visualized using UV light.

## Results

**Figure 1.**
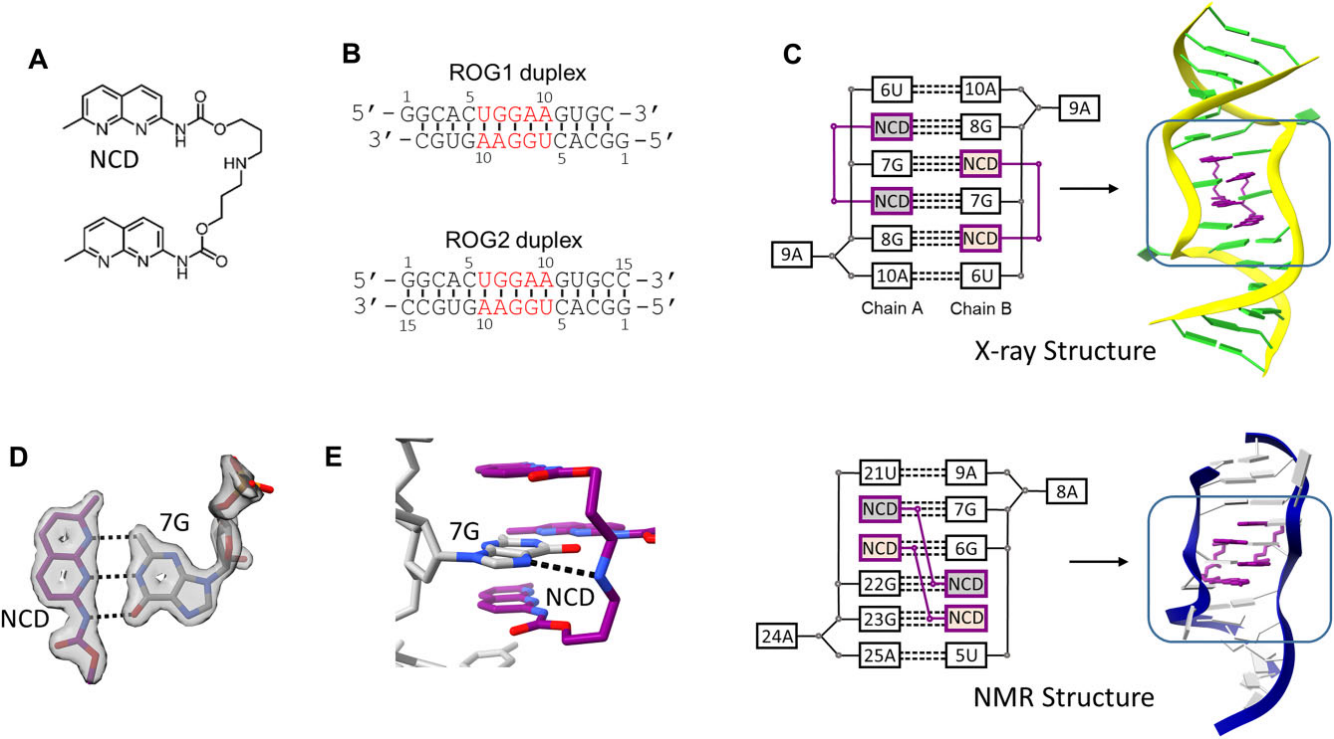
Interactions between NCD ligand and RNA. (**A**) Chemical structure of NCD molecule. (**B**) Duplexes of ROG1 and ROG2 oligomers (the UGGAA motif is presented in red). (**C**) Secondary structure diagrams and tertiary structures of interactions between the UGGAA motif and NCD molecules (purple) in X-ray crystallographic (upper part) and NMR (lower part) models. (**D**) Pseudo-canonical base pair formed between the NU of NCD and the guanosine residue. The 2F_o_–F_c_ electron density map (gray) is contoured at the 1σ level. H-bonds are represented by black dashed lines. (**E**) H-bond between the amino group of the NCD linker and the imino group of the neighboring G residue.

In this study, we used a NCD ligand containing two naphthyridine units (NU) connected by a relatively long aliphatic linker (3-(carbamoyloxy)propylaminopropyl carbamate) consisting of eleven atoms (Fig. 1A). The distribution of nitrogen atoms in NU was designed to form base pairs with the guanosine residues [15, 48, 49]. As a result the NCD was found to selectively bind two guanosines located in single-stranded or bulged regions of nucleic acids [50, 51]. Based on a previous study, we designed and synthesized two RNA oligomers (ROG1 and ROG2). Each of the oligomers formed self-complementary duplex: ROG1 (GGCACUGGAAGUGC)_2_ and ROG2 (GGCACUGGAAGUGCC)_2_; containing the 5′-UGGAA-3′/5′-UGGAA-3′ pentad motif selectively recognized by NCD (Fig. 1B) [17, 52]. ROG1 was shorter than ROG2 by one cytosine residue at the 3′ end. We crystallized two RNA–ligand complexes: ROG1–NCD (1.50 Å resolution) and ROG2–NCD (2.55 Å resolution) and one unliganded duplex of ROG2 oligomer (2.79 Å). All X-ray data and refinement statistics are presented in Supplementary Table S1. The electron density maps of all the crystal models were unambiguous resulting in well-defined positions and conformations of the RNA and NCD ligands (Supplementary Fig. S1).

### General overview of RNA–ligand structures

In both liganded structures, the asymmetric unit contained one RNA duplex consisting of chains A and B. The superposition of the ROG1–NCD and ROG2–NCD models showed similarity, with an r.m.s.d (root mean square deviation) value of 0.8 Å. The highest conformational similarity was observed in the central part of the structures embedding the 5′-UGGAA-3′/5′-UGGAA-3′ motif (r.m.s.d was 0.67 Å of main chain). The pentad motif bound two NCD molecules that interacted with all four guanosine residues (Fig. 1C). As a result, six base pairs were formed: two 6U–10A pairs and four pseudo-canonical naphthyridine-guanine pairs. The 9A residues were flipped out and engaged in crystal lattice contacts with symmetry-related RNA molecules (Fig. 1C and Supplementary Fig. S2). The pentad motif showed structural symmetry, as reflected by identical interactions between the ligand and guanosine residues.

In the ROG1–NCD complex, a third ligand molecule was observed between the 5′ ends of the symmetry-related helices, participating in crystal lattice formation (*vide section: Role of NCD ligand in crystal lattice formation*).

### Interactions between NCD and UGGAA motif

The NCD molecules were located in the minor groove of the 5′-UGGAA-3′/5′-UGGAA-3′ motif. Each ligand bound two guanosine residues located on the same RNA strand, forming identical pseudo-canonical NCD-G pairs (Fig. 1C). Each NU unit interacted with the guanine base *via* three hydrogen bonds (Fig. 1D). The first hydrogen bond was formed between the N1 imino group of naphthyridine and the N2 amine group of guanine. A second bond was formed between the N8 imino group of NU and the secondary nitrogen atom N1 of the guanine amino group. A third interaction occurred between the amide group of the NU moiety and O6 carbonyl of the G base. Additional RNA–ligand interaction was observed between the amine group of the NCD linker and the N7 imine group of the G7 residue (Fig. 1E). All four pseudo-canonical base pairs formed a stack consisting of alternating pairs: NCD-G8, G7-NCD, NCD-G7, G8-NCD. This arrangement resulted in the location of G7 residues from chains A and B between two NU units from the same ligand molecule (Fig. 1C). Likewise, one of the NU units was located between the U6 and G7 residues, and the second NU unit was located between the G7 and G8 residues. In other words, the U6, G7, and G8 residues from each RNA strand were separated by wedged NU units of NCD ligands, increasing the distance between neighboring residues from the typical value of approximately 3.2 Å to approximately 6.5 Å. Despite the formation of pseudo-canonical pairs, only limited stacking interactions between the naphthyridines and nitrogen bases were observed.

### Role of NCD ligand in crystal lattice formation

**Figure 2.**
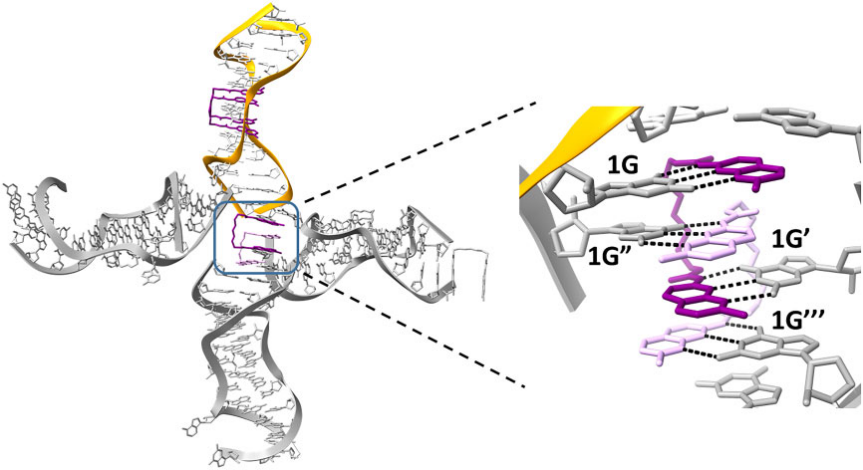
Crystal lattice formation by NCD ligand. Two ligand molecules, NCD (dark purple) and NCD’ (light purple), bind four guanosine residues of four symmetry-related RNA molecules.

In the ROG1–NCD model, the third ligand molecule connected the symmetry-related RNA helices. The contacts in the crystal lattice were formed by two NCD molecules (one from the asymmetric unit and second, NCD’, from the symmetry-related complex) and four RNA duplexes (one from the asymmetric unit and three from symmetry-related RNAs) (Fig. 2). The NCD was bound to the 1G residue of chain B and 1G of chain A’. Similarly, the NCD’ linked the 1G residue of chain B’’ and 1G of chain A’’’. In total, four pseudo-canonical G-NCD pairs were observed, and similar to the 5′-UGGAA-3′/5′-UGGAA-3′ motif, they were arranged in an alternating manner (the NCD-G pairs were separated by symmetry-related NCD’-G pairs) (Fig. 2). The observed lattice interactions resulted in the alignment of two helices, one above the other, while the other two helices were perpendicular to the RNA helix from the asymmetric unit. In ROG2 and ROG2–NCD models, lattice contacts were formed by stacking interactions between symmetry-related duplexes, resulting in the formation of pseudo-continuous helices.

NCD was shown earlier to act as a molecular glue for DNA and RNA molecules [19–22]. To further investigate the properties of NCD in facilitating the interaction between ROG1 duplexes in solution, we performed native gel electrophoresis of ROG1 RNA in the presence of increasing concentrations of NCD ligand (Supplementary Fig. S3A). A slower migrating bands were observed within a range of NCD:ROG1 ratio of 1.8:1–30:1, indicating the formation of higher-order NCD–ROG1 complexes. At the two highest NCD concentrations, the intensity of all bands was greatly reduced suggesting the NCD-dependent RNA aggregation.

### Ligand conformation

**Figure 3.**
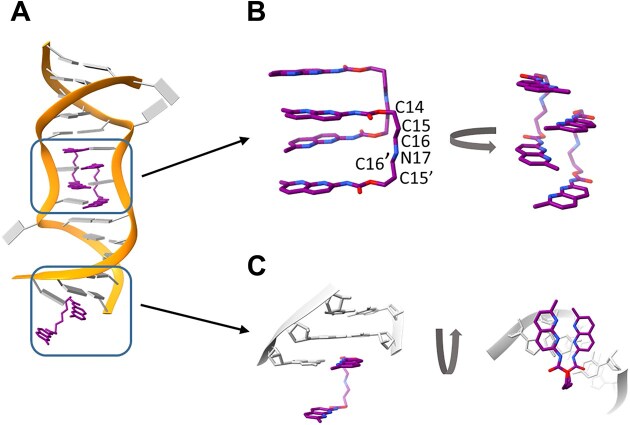
Conformation of ligand molecules in the ROG1–NCD structure. (**A**) Binding sites of the NCD in the ROG1 helix. (**B**) Conformation of the NCD molecules that bind to the UGGAA motif. (**C**) Structural arrangement of the NCD participating in the crystal lattice contacts formation.

All ligand molecules exhibited a square bracket-like conformation. The aliphatic part of the linker was elongated, whereas the NUs with carbamate (R-NH-CO-O-R’) groups were perpendicular to the linker (Fig. 3A). In the case of NCD ligands bound to the pentad motif, the aliphatic part of the spacer consisted of six atoms (C14, C15, C16, N17, C16’, and C15’). They were arranged in a zigzag line with a length of 6.3 Å. The NU units of each NCD ligand were parallel and stacked one above another (Fig. 3B). Moreover, the nitrogen atoms of NU were oriented in the same direction (*cis* geometry).

In the ROG1–NCD model, the aliphatic linker of the ligand connecting symmetry-related molecules showed a similar zigzag conformation but consisted of seven atoms (C14, C15, C16, N17, C16’, C15’, and C14’). This resulted in a longer distance (7.4 Å) between the two NU planes. Additionally, the NUs were twisted relative to each other by 17° and their nitrogen edges showed a trans geometry (Fig. 3C).

### Unliganded RNA duplex structure

The unliganded ROG2 oligomer crystallized as a self-complementary duplex. Similar to the ROG2–NCD structure, the 5′-UGGAA-3′/5′-UGGAA-3′ motif was located in the central part of the helix and flanked by five Watson–Crick base pairs.

**Figure 4.**
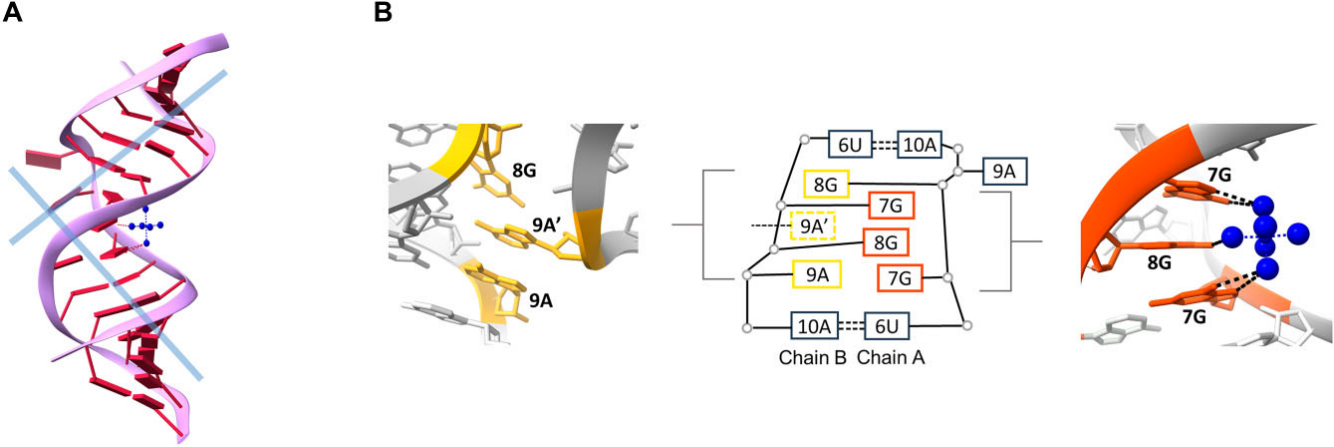
Unliganded structure of the ROG2 duplex. (**A**) The bending of the helix is marked by blue lines representing helical axes. The hexammine cobalt cation (blue) is located in the major groove. (**B**) 3 × 3 internal loop formed by the UGGAA motif. One of the nucleotides stack (yellow) is located in the minor groove while the second (orange) in the major groove. Hexammine cobalt cation (blue) interacts with guanosine residues of 3 × 3 internal loop.

The distal parts of the helix exhibited an A’-RNA form, with nucleotides in the C3’-endo or C2’*-exo* conformation (Fig. 4A). The values of helical parameters were within A’-RNA range: Zp > 1.5°, twist = 31.8°, and rise = 3.2Å [53–55]. The GGA residues of the pentad motif formed an internal 3 × 3 loop (GGA/AGG) showing double-stranded character in terms of strand separation, but deviated from the A-RNA helix, which was reflected in most of the helical parameters (e.g. stretch, twist, or rise) (Supplementary Table S2). Moreover, the presence of an internal loop was associated with a bending of the helix by 100° (Fig. 4A). In contrast to the liganded structures, the arrangement of the pentad motif residues in the unliganded model was not symmetric. It consisted of two flanking 6U–10A Watson–Crick base pairs and two cross-strand stacks of nucleotides. The first stack was located in the major groove of the helix and consisted of three guanosine residues: 7G and 8G from chain B, and 7G from chain A (Fig. 4B). The second stack, located in the minor groove, consisted of 9A from chain B, 9A’ from symmetry-related chain A’, and 8G from chain A (Fig. 4B and Supplementary Fig. S4). Both stacks could be extended by neighboring U-A pairs. The 6U from chain A overlapped with the first stack, whereas 10A from chain B overlapped with the second stack. The conformation of the nucleotides in the first stack was typical for the A-RNA form. In the second stack, the 9A exhibited a *syn* conformation. The 9A from chain A’ had C2’-endo sugar puckering and an *anti* conformation of the nucleobase. The remaining 8G was shifted toward the minor groove and showed a high *anti-conformation*, reflected in the χ torsion angle having −104.2° value instead of approximately 180° [55].

The cross-strand stacks of nucleotides were almost parallel to each other and formed only one H-bond between nucleobases (*exo*-amino group of 7G in chain B and the N7 imino group of 8G in chain A). Other interactions were observed between the guanine nucleobases and phosphate backbone (Supplementary Fig. S5). Additionally, the guanosine residues of the first stack interacted with the cobalt hexammine [Co(NH_3_)_6_]^3+^ cation located in the major groove of the helix (Fig. 4).

### Structural changes of RNA associated with ligand binding

The comparison between the unliganded ROG2 and the ROG1/ROG2–NCD models showed that the flanking base pairs of the pentad motif were similar in terms of helical parameters (Supplementary Table S3). The superposition of the flanking base pairs of ROG2 and ROG2–NCD showed r.m.s.d. of 0.3 Å for residues 11–15 of chain A and residues 1–5 of chain B and r.m.s.d. of 0.9 Å for residues 1–5 of chain A and 11–15 of chain B. Major differences between the unliganded and liganded models were observed for the 5′-UGGAA-3′/5′-UGGAA-3′ motif. Only the flanking U-A pairs remained the same, whereas other residues underwent substantial rearrangement. In the liganded models, the pentad loop was unwound, resulting in loss of helicity (Fig. 1C). Both 9A residues were flipped out, while guanosines interacting with the NCD ligands formed a double-stranded stem resembling the Z-RNA like structure [56] (Supplementary Fig. S6). The sugar-phosphate backbone followed a zigzag course, which was reflected in α (O3′—P—O5′—C5′) and ζ (C3′—O3′—P—O5′) torsion angles of 7G and 8G residues of both RNA strands (Supplementary Table S4). This unusual conformation resulted in a large distance between the 6U, 7G, and 8G residues (6.4–7.0 Å). The resulting gaps were filled with the naphthyridine rings of the NCD ligands. As in Z-RNA form, guanosines of the pentad motif had *syn* conformation of the N-glycosidic bond and alternating sugar puckering from C3’-endo for the 7G residue to C2’-endo for the 8G residue. However, although the pentad motif resembled many structural features of the Z-RNA form, the structure remained right-handed, as in A-RNA (Supplementary Fig. S6).

### Physicochemical data

**Figure 5.**
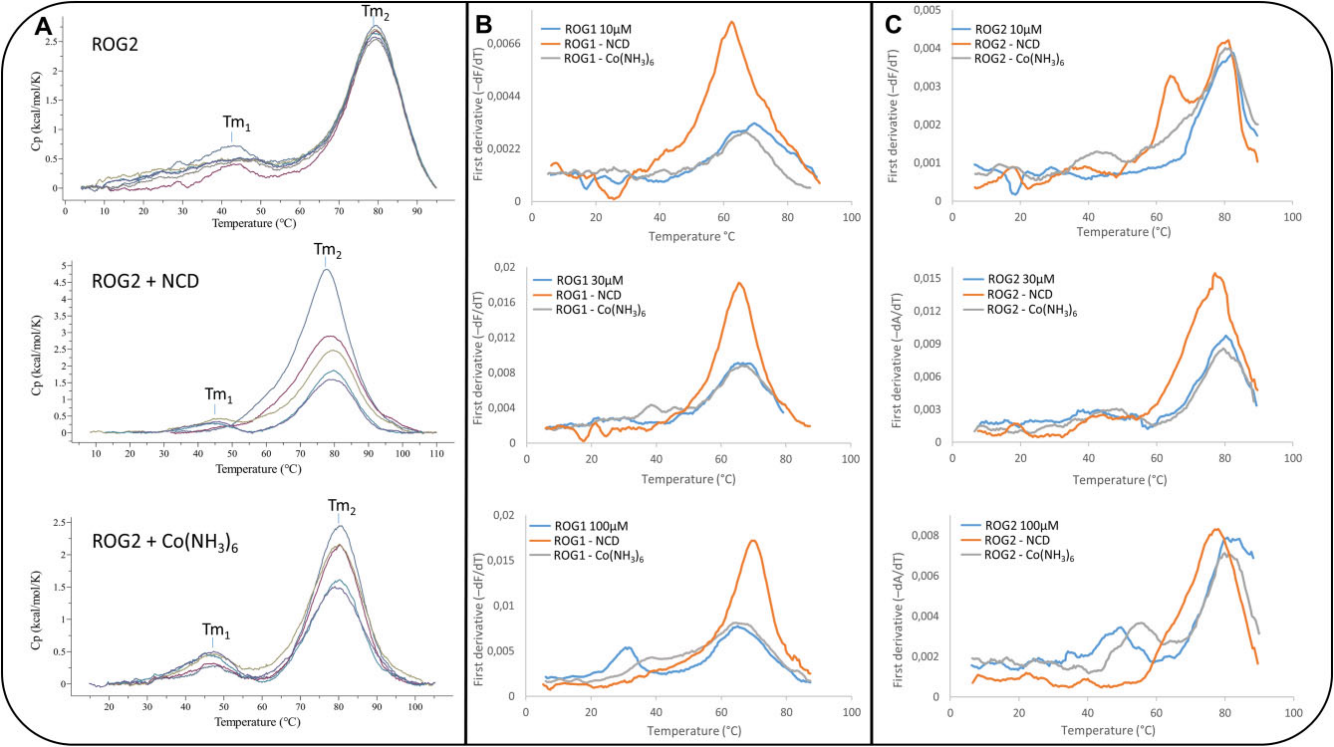
Physicochemical evaluation of ROG1 and ROG2 duplexes and their interactions with NCD and Co(NH_3_)_6_ ligands using (**A**) DSC and (**B**, **C**) thermal denaturation monitored by UV spectroscopy. The first derivative of absorption (−dA/dT) was plotted against temperature.

DSC was used to determine the thermal properties of the RNA samples. Since the ROG2 oligomer contained one binding site for NCD molecules, it was used for DSC evaluation. Three samples were measured: unliganded ROG2, the ROG2–NCD complex, and ROG2 in the presence of hexamminecobalt [ROG2-Co(NH_3_)_6_], which was present in the crystal structure. In general, the DSC spectra of all the samples in all cycles showed two peaks (Tm1 and Tm2) (Fig. 5A). The Tm1 peak was observed at 43°C for unliganded ROG2, 43°C for ROG2–NCD and 46°C for ROG2-Co(NH_3_)_6_ complex after the third round of denaturation–renaturation cycle. The Tm2 peak was observed at nearly the same temperature (79.0°C), regardless the absence or presence of the ligand molecule (Supplementary Table S5A). In the case of the ROG2–NCD complex, the first and second round of denaturation–renaturation cycle resulted in one major peak which was higher and slightly asymmetric than those of the other samples. Almost no decay of signal was observed for unliganded ROG2 across all five rounds of denaturation–renaturation cycle, whereas in the presence of hexamminecobalt and NCD gradual diminishing of the peak was observed (Fig. 5A). To determine whether the decay of the signal was associated with RNA degradation, we performed denaturing gel electrophoresis of the samples after DSC measurements. The DSC ROG2–NCD band was diffused in comparison to the control line, whereas for DSC ROG2-Co(NH_3_)_6_, it was well-defined (Supplementary Fig. S3B). The diffused band observed for the ROG2–NCD sample suggests temperature-dependent NCD decomposition leading to disruption of RNA–ligand complex. This is reflected in the DSC spectra because after the third cycle, the DSC profile resembles that of the unliganded ROG2 duplex.

To compare the thermal stability of the ROG1 and ROG2 duplexes, we used the UV-melting method (Fig. 5B and C and Supplementary Table S5B). The measurements were performed for ROG1, ROG2, and their complexes with [Co(NH₃)₆]³⁺ and NCD ligands at three different RNA concentration (10, 30, and 100  μM) to assess its influence on RNA melting behavior. At 100  μM RNA, the UV melting curves of unliganded ROG1 and ROG2 exhibited two distinct peaks. The first peak occurred at approximately 33°C for ROG1 and 48°C for ROG2. At lower RNA concentrations (10 and 30  μM), the first transition was difficult to resolve due to its low energetic contribution, resulting in a broad and shallow peak. In contrast, the second peak was well defined at all RNA concentrations, occurring at approximately 67°C for ROG1 and 80°C for ROG2. In the presence of [Co(NH₃)₆]³⁺ (at 100  μM RNA), a shift in the first peak was observed (Δtm = 6°C for ROG1 and Δtm = 7°C for ROG2). In general, UV melting of the RNA–NCD complexes revealed a single, asymmetric peak (at 65°C for ROG1–NCD and 77°C for ROG2–NCD). The only exception was observed for 10 μM ROG2–NCD, where two transitions appeared: the first at ∼62°C and the second at ∼80°C (Fig. 5C and Supplementary Table S5B).

To exclude the influence of the RNA concentration on the RNA heterogeneity we performed native gel electrophoresis. The results clearly shows high structural homogeneity of both duplexes regardless the RNA concentration (Supplementary Fig. S3C).

Structural rearrangements in ROG2 upon ligand titration were investigated using CD (Supplementary Fig. S7). The most significant changes in the CD spectra were observed in the 245–225 nm range. The binding of NCD induced an inversion of the peak at 233 nm from a negative to positive value. Other spectral changes were observed at 263 nm for the decreasing peak and in the 355–325 nm range.

## Discussion

### NCD binding induces Z-RNA-like conformation

The crystal model of the unliganded ROG2 oligomer allowed the tracking of the structural rearrangements of RNA upon NCD binding, which was not possible in an earlier study [17]. The conformational changes were significant and mostly involved the 5′-UGGAA-3′/5′-UGGAA-3′ motif. In the native structure, it formed an internal loop consisting of two flanking U-A pairs and two stacks of nucleotide residues. One consisting of three guanosines and the second consisting of two adenosines and one guanosine (Fig. 4B). Although the stacked nucleotides were not engaged in base pairing, they were well defined and ordered. The helical character of the duplex was maintained; however, the major groove was narrowed and the helix was bent (Fig. 4A). Binding of NCD molecules resulted in unwinding of the pentad motif and stretching of the helix, similar to the Z-RNA form (Supplementary Fig. S6). All guanosines of the pentad motif switched from *anti* to *syn* conformation and formed H-bonds with the NUs of the ligand, whereas 9A residues from both strands were flipped out. Consequently, the pentad motif gained internal symmetry, which was not observed in the unliganded structure.

Structural changes of RNA were also observed in the CD spectra. The inversion of the peak at 235 nm from negative to positive upon ligand saturation could indicate unfolding of the pentad motif, leading to the loss of helicity (Supplementary Fig. S7). The slight changes in the shape of the spectra in the 325–355 nm range were most likely related to the chirality of the NCD molecule. Upon binding to RNA the achiral NCD became the chiral component of the complex. A similar effect was observed in our previous study [8].

The biological impact of NCD binding to RNA containing r(UGGAA)_n_ was observed in HeLa cells and a Drosophila model of SCA31 [17, 49]. HeLa cells expressing toxic r(UGGAA)_76_ RNA and exposed to the NCD ligand exhibited a significant reduction in RNA foci formation while in the Drosophila, NCD showed suppression of disease phenotype. NCD’s binding to mutated transcripts can either block RNA-binding protein (RBP) sites or alter the RNA structure so it is no longer recognized by RBPs. It is conceivable that r(UGGAA)_76_ forms a hairpin structure with a long stem composed of U-A and A-U pairs interrupted by GGA internal loops. Based on our results NCD binding stabilizes stem structure by forming a paired region with the guanosine residues of the GGA motif, inducing a conformational change in the RNA from an A to a Z-like form. Consequently, the internal loop becomes unavailable for interactions, and the global RNA structure differs from its unbound state. This can explain why in the presence of NCD ligand r(UGGAA)_n_ repeats are no longer a toxic factor in SCA31 pathogenesis in model organism [17]. The family of mismatch binding molecules (MBL) compounds, including NCD, can induce structural rearrangement of RNA [9–11, 17, 50]. The crystallographic and NMR structures of complexes of nucleic acids with other ligands such as naphthyridine-azaquinolone molecules and cyclic mismatch-binding ligands showed that ligands formed pseudo-canonical base pairs with target nucleotides [11, 14, 16, 57]. As a consequence, the neighboring residues were flipped out in order to fit into the structural changes of the DNA/RNA helix imposed by ligand binding. We observed that NCD binding caused an unexpected loss of helicity, which would be difficult to predict using *in silico* approaches. Hence, the presented structures can serve as excellent reference models for the evaluation and improvement of existing and development of future approaches for computational prediction of RNA–ligand interactions.

### Comparison of crystal structures with the NMR model

In a previous study, the NMR structure of the RNA–NCD complex has been described [17]. Similar to the crystallographic models, two NCD molecules were bound to guanosine residues of the 5′-UGGAA-3′/5′-UGGAA-3′ motif. Both adenosine residues were flipped out and two flanking A-U pairs were formed (Fig. 1C). However, the arrangement of the NCD molecules in the NMR model was different from that in the X-ray structures. Each ligand molecule was base-paired with guanosine residues located on opposite RNA strands. As a result, the G residues involved in ligand binding were not separated by the NUs. Consequently, two naphthyridines from each ligand were adjacent and located between the uridine and two consecutive guanosines (Fig. 1C). The conformations of the NCD linkers also differed, representing a disordered C-shape rather than an ordered zigzag-like conformation and the amino group of the linker did not form H-bond with N7 imino group of guanosine residue as in X-ray model (Fig. 1E).

It is difficult to explain the distinct folding of the RNA–NCD complex in the NMR and crystallographic structures. Crystallographic models were determined based on electron density maps, providing unambiguous information on the atomic positions of the NCD and RNA molecules and eliminating other binding possibilities. The NMR model with neighboring G6–G7 and G22–G23 guanosine residues was based on the observation of weak NOE signals of exchange of the imino protons of guanosine residues, suggesting their close proximity (Fig. 1C) [17]. Using NMR restraints, only the arrangement of neighboring guanosine residues resulted in reasonable structures. The existence of two modes of NCD binding could arise from the kinetic or thermodynamic effects present in the experimental conditions. In solution, a starting point for both crystallographic and NMR experiments, the internal loop of the UGGAA motif probably shows structural dynamics. Thus, the initiation of NCD binding can influence the subsequent events during the full complex formation. In one possible scenario, the interactions between the ligand and RNA could start with the formation of H-bonds between one NU and one of the guanosine residues, most likely 8G from chain B located in the minor groove (Fig. 4B). The formation of interactions between the second NU and second G residue on the same or opposite strand would lead to the formation of two different conformations of the RNA–ligand complexes. When second NU binds the G residue on the opposite strand, an NMR-like structure will be observed. When the second guanosine is located on the same strand, the complex will form crystallographic-like structure. The formation of an NMR-like structure may be a kinetically driven process, whereas the crystallographic structure is thermodynamically dependent. Over time, the kinetic product of the RNA–ligand complex can be converted to a thermodynamically stable structure due to partial dissociation of ligand and formation of crystallization-like interactions. The RNA–ligand complexes were prepared under similar conditions for NMR and crystallography analysis [20 mM sodium phosphate (pH 6.9) and 100 mM NaCl for NMR and 10 mM sodium cacodylate (pH 7.0) and 100 mM NaCl for crystallography]. However, for crystallization, the RNA–ligand complexes were further mixed with a buffer containing precipitants such as 45% MPD and 1.3 M lithium sulphate. This may change the balance between the kinetic and thermodynamic products compared to the NMR experiment. Alternatively, both types of complexes are formed in solution but usually only one of the conformational state forms crystals while in NMR spectra overlapping signal of two conformers could be difficult to distinguish [58].

### The architecture of the internal loop

The unliganded structure of ROG2 contained a purine (GGA/AGG) 3 × 3 internal loop, in which nucleotides were unpaired and formed two cross-strand stacks of residues (Fig. 4B). The search using RNA FRABASE revealed that the GGA/AGG loop was not observed in RNA structures deposited in the PDB database [59]. Instead we found three other types of 3 × 3 purine-rich loops: GAA/AAG, GAA/AGG, and GAG/AGG. Unlike in the loop of ROG2 RNA, their residues were paired and formed noncanonical G-A, A-A, and G-G pairs. However, using the RNA 3D motif atlas, we identified a 3 × 3 unpaired loop with structural characteristics similar to those of the loop in ROG2 RNA. The loop was located in archaebacterial rRNA from the large ribosomal subunit (LSU) [60, 61]. Three adenosine and cytosine residues formed one cross-strand stack, whereas guanosine and cytosine formed a second stack (Supplementary Fig. S8). In general, the unpaired 3 × 3 purine loop can affect the structure and function of RNA molecules in many ways by: (i) maintaining the double stranded character of RNA in terms of stacking interactions, (ii) inducing of bending of RNA helix, (iii) exposing nucleobases to the solvent moiety, (iv) involving in tertiary and quaternary interactions, or (v) serving as a ligand-binding site. In the rRNA of LSU, the two stacked adenosine residues were engaged in interactions with the neighboring RNA helix, whereas in ROG2, the residues of the 3 × 3 loop were either involved in the crystal lattice contacts and/or bound ligand molecules (NCD or [Co(NH_3_)_6_]^3+^).

### Local stabilization of the RNA structure by NCD and cobaltamine molecules

The binding of cobaltammine and NCD molecules has influence on the thermal stability of the internal loop of RNA. The UV-melting and DSC spectra of unliganded ROG1 and ROG2 showed two transition points (Fig. 5). The first peak corresponds to the melting of the internal UGGAA loop, while the second peak represents the denaturation of the double-stranded region of the duplex. The shift of the first peak observed in the presence of [Co(NH₃)₆]³⁺ supports its role in local stabilization of the internal UGGAA loop. This interpretation is consistent with crystallographic data of the unliganded ROG2 oligomer, which shows direct interactions between [Co(NH₃)₆]³⁺ and the guanosine residues of the UGGAA motif. In the presence of the NCD ligand, the first peak was no longer distinct while the second peak became asymmetric in the UV and DSC profiles. These alterations in the melting behavior imply that the first transition was shifted and merged with the second, indicating that NCD locally stabilizes the internal loop. In case of UV profile at the 10 μM ROG2–NCD concentration, the peaks were not merged. This can be explained by the fact that the melting point of RNA duplexes is concentration-dependent leading to the migration of the peaks [62].

### NCD exhibits a molecular glue characteristics

Our previous studies showed that NCD and NCD derivatives serve as molecular glue for the assembly of DNA molecules into large tetrahedron structures and modulation of the function of structured RNAs (ribozymes, riboswitches, and pseudoknots) [19–22]. In the present study, we showed that this property of NCD can be applied for crystal lattice formation. In the ROG2–NCD model, two NCD ligands bridged four neighboring RNA molecules into contact, leading to the formation of a crystal lattice in all directions. NCD is a promising molecular tool because it exhibits several important modalities. First, it possesses two selective and specific RNA binding sites for unpaired guanosine residues, forming strong and direct interactions with the target RNA. Second, the NCD linker is not only a passive element connecting NUs but also an important contributor to the selectivity and specificity of the ligand. It prefers the elongated zigzag conformation and has the ability to form hydrogen bonds with RNA having an amino group (Fig. 1E). The zigzag conformation results in a linker length of ∼7 Å, which is exactly double the value of the rise parameter corresponding to the distance between the neighboring bases in the RNA helix. Therefore, the gap between the two NCD-G pairs could be filled by the NCD-G pair of the second ligand. This observation is in agreement with recent results aimed at optimizing the linker length [63]. The longer linker resulted in weaker NCD binding, while the reduction of one atom (from 11 to 10 atoms) caused stronger binding than the parental NCD molecule. In the structure of ROG1–NCD, the zigzag line of the linker contains six or seven atoms, suggesting that the removal of one atom could limit the conformational freedom of the linker and increase the affinity of the ligand. Third, NCD prefers a tandem mode of binding, which means that exclusively two NCD molecules interact with RNA molecules. The resulting four NCD-G pairs alternate, and this arrangement is stabilized by interactions of the amino groups of the NCD linkers with the N8 imino group of guanosines. Fourth, the protonation state of NCD remains stable under neutral, slightly acidic, and slightly basic pH conditions, indicating that the ligand maintains its binding properties in a physiological environment. Due the presence of the carbamate group (R-NH-CO-O-R’), under strongly acidic or basic conditions, the molecule undergoes decomposition.

The crystallization of RNA molecules is challenging. Successful crystallization depends on the proper design of the RNA sequence which will induce contacts between the RNA molecules and crystal lattice formation. One of the methods to enhance RNA crystallization was to use 5′ or 3′ overhanging nucleotides that base-pair with the other end of the neighboring RNA molecule forming pseudo-infinite helices [64]. The use of NCD as a “molecular glue” for crystal lattice formation may be more advantageous because it connects four RNA molecules instead of two, as in the case of overhanging nucleotides. This resulted in formation of crystal lattice in all directions and improved the data resolution from 2.55 Å (ROG2–NCD) to 1.5 Å for ROG1–NCD. We believe that the exploitation of the NCD in RNA crystallography could contribute to determining the structures of many functional RNAs, increasing our knowledge of RNA structure and function relationship.

## Supplementary Material

gkaf924_Supplemental_File

## Data Availability

Atomic coordinates and structure factors for the reported crystal structures have been deposited with the Protein Data Bank under accession numbers 9IF1, 9IF0 and 9I9W. Diffraction images have been deposited in Macromolecular Xtallography Raw Data Repository (MX-RDR, mxrdr.icm.edu.pl): doi:10.60884/QV3IGO for ROG2 RNA, doi:10.60884/NO2MXW for ROG2–NCD complex and doi:10.60884/SCBCTX for ROG1–NCD complex.

## Supplementary data

Supplementary data is available at NAR online.
