# Supplementary material for: Naphthyridine carbamate dimer ligand induces formation of Z-RNA-like fold of disease-related RNA and exhibits a molecular glue characteristics in crystal lattice formation
Source: Nucleic Acids Res. 2025 Sep 17;53(17):gkaf924. doi: 10.1093/nar/gkaf924 (PMC12448875; doi:10.1093/nar/gkaf924)
Supplement: gkaf924_Supplemental_File [file gkaf924_supplemental_file.pdf]

Naphthyridine carbamate dimer ligand induces formation of Z-RNA-like fold of disease-related RNA and exhibits a molecular glue characteristics in crystal lattice formation

Martyna Mateja-Pluta[1], Leszek Błaszczyk[1],Magdalena Bejger[1], Kazuhiko Nakatani[2],

Agnieszka Kiliszek[1]*

[1] Institute of Bioorganic Chemistry, Polish Academy of Sciences, Z. Noskowskiego 12/14, 61-704, Poland

[2] Department of Regulatory Bioorganic Chemistry, SANKEN (The Institute of Scientific and Industrial Research), Osaka University, 8-1 Mihogaoka, Ibaraki, 567-0047, Japan

* To whom correspondence should be addressed. Tel: +48 61 852 8503 ext. 1278; Fax: +48 61 852 05 32; Email: kiliszek@ibch.poznan.pl

	ROG2	ROG2-NCD	ROG1-NCD
**Data Collection**			
X-ray source	Eiger1 X 4M Mx 2432	PILATUS 6M	Dectris PILATUS 6M-F,
Space group	$P4_12_12$	C121	$P4_12_12$
Cell parameters (Å)	a = 94.46, b = 25.31, c = 45.41	a = 94,46, b = 25.31, c = 45.41	a = 71.63, b = 71.63, c = 62.88
Resolution (Å)	42.52-2.79 (2.96-2.79)	46.11-2.55 (2.7-2.55)	47.3-1.5 (1.59-1.5)
R_{merge}	0.059 (0.364)	0.066 (1.159)	0.085 (1.792)
I/σ	16.72 (2.64)	16.17 (1.73)	24.64 (1.91)
$CC_{1/2}$	0.999 (0.982)	0.99 (0.844)	1.0 (0.89)
Completeness (%)	97.3 (82.3)	98.4 (98.9)	100 (100)
Redundancy	6.26 (5.84)	6.03 (6.08)	25.7 (24.9)
Number of unique reflections	2764	3544	26777
**Refinement**			
Software	Refmac 5.8.0258	Refmac 5.8.0258	Refmac 5.8.0258
Number of reflections: work/test	2485/277	3358/185	25879/899
Overall mean B value ($Å^2$)	28.256	65.69	26
R_{work}/R_{free}	0.2625/0.3085	0.201/0.2397	0.129/0.166
RNA atoms	656	725	1017
Water molecules	6	6	203
Ligand molecules	NCO	NCD	NCD
RMSD in bonds (Å)	0.009	0.008	0.012
RMSD in angles (º)	1.742	1.836	1.616
PDB code	9IF1	9IF0	9I9W
x-ray images	10.60884/QV3IGO	doi:10.60884/NO2MXW	doi:10.60884/SCBCTX

Supplementary Table S1: Summary of X-ray data and model refinement statistics.

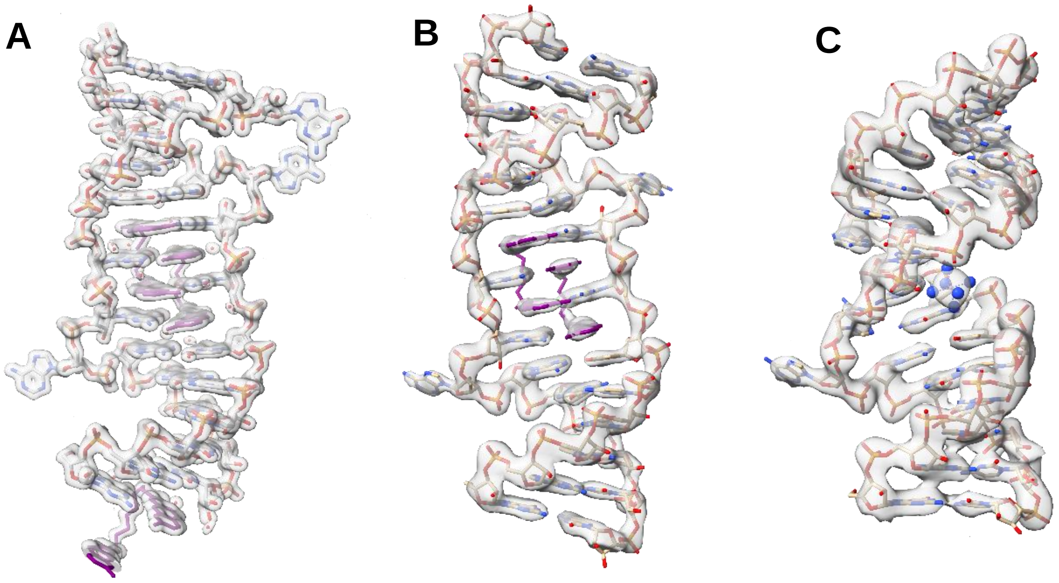

Supplementary Figure S1: The $F_o - F_c$ omit map of (A) ROG1-NCD, (B) ROG2-NCD and (C) ROG2 contoured at the σ 1.0 level.

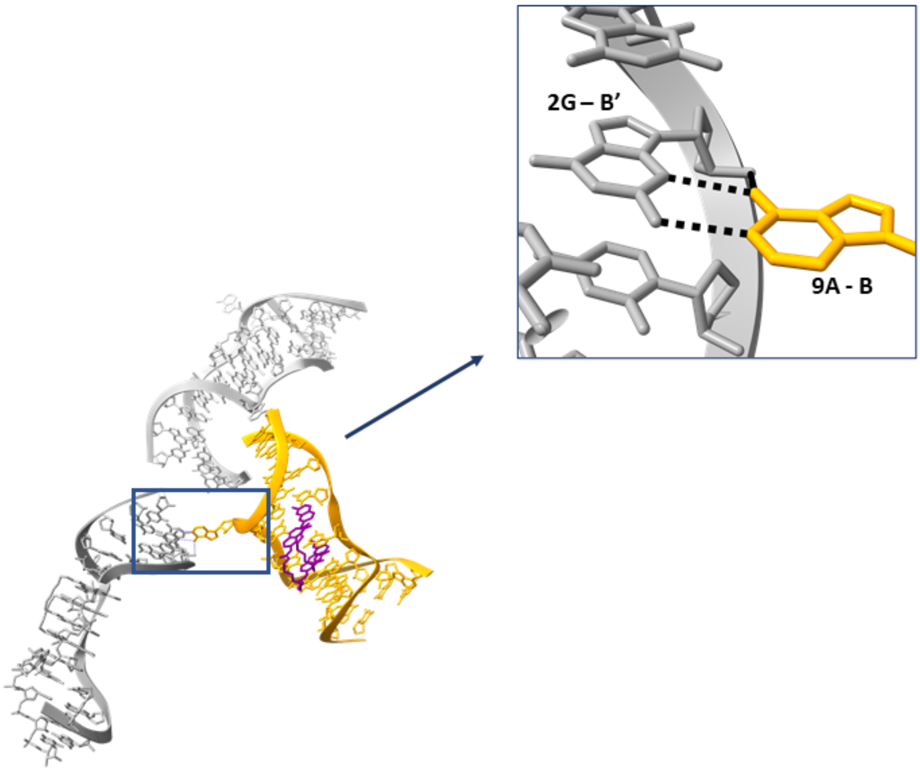

Supplementary Figure S2: The interactions between the flipped out 9A residue forming crystal lattice contacts in ROG1-NCD. The H-bonds are represented by black dashed lines.

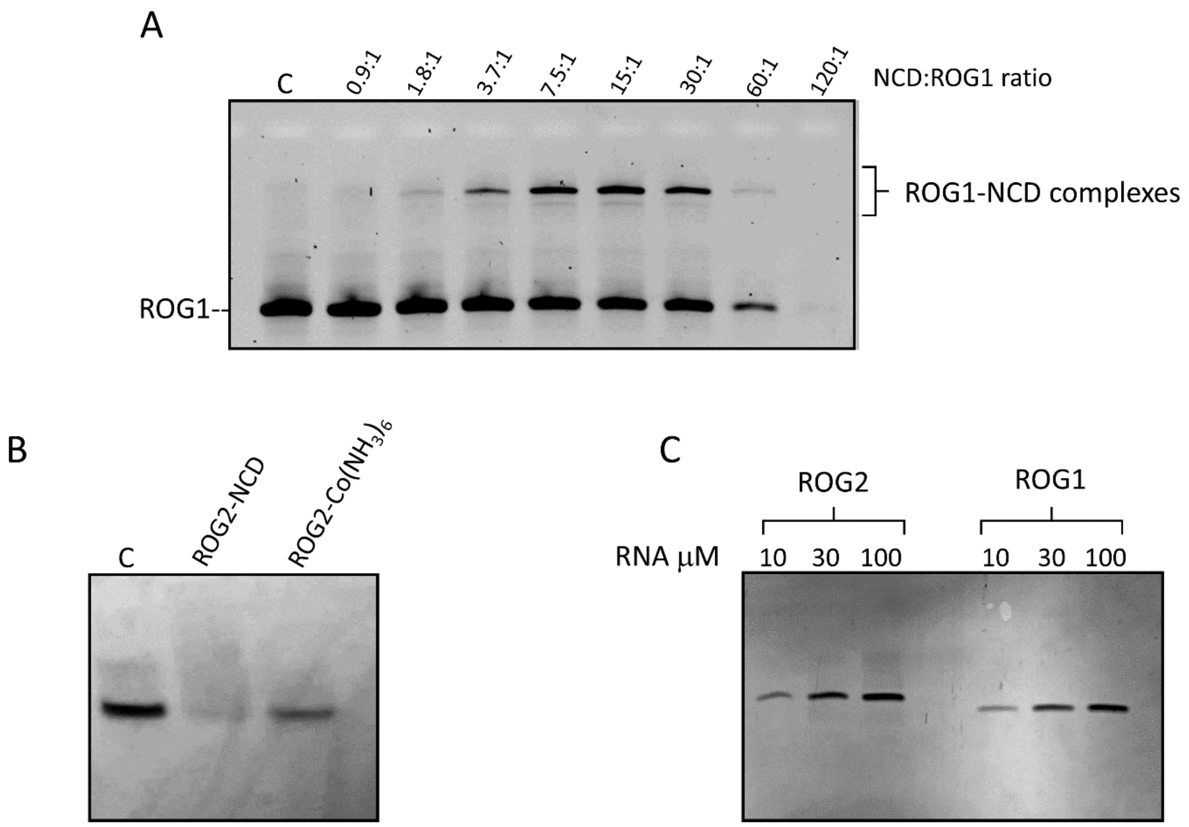

Supplementary Figure S3: Native and denaturing gel electrophoresis of RNA and RNA-ligand complexes. (A) Electrophoretic mobility shift assay of ROG1-NCD complexes. C – control reaction (unliganded ROG1 duplex). (B) Denaturing gel electrophoresis of ROG2-NCD and ROG2-Co$(NH_3)_6$ complexes after DSC measurements. C – control reaction (unliganded ROG2 duplex). (C) Native gel electrophoresis of ROG2 and ROG1 oligomers in three concentrations: 10, 30 and 100 μM.

ROG2						
bp	Shear	Stretch	Stagger	Buckle	Propeller	Opening
1 G-C	-0,58	-0,18	0,18	-10,07	-15,38	3,22
2 G-C	0,02	-0,14	-0,05	-8,74	-14,88	-4,75
3 C-G	-0,03	-0,21	-0,09	4,8	-11,28	2,77
4 A-U	-0,06	-0,23	0,58	3,75	-10,31	-5,4
5 C-G	0,16	0	-0,1	2,95	-21,01	1,18
6 U-A	0,91	-0,01	0,58	-9,36	-7,9	2,86
* 7 G-G	6,75	3,89	0,16	37,3	-31,25	-59,45
* 8 A+G	4,33	4,39	0,37	-7,65	13,6	104,46
9 A-U	-0,34	-0,09	-0,29	-9,22	4,95	-6,6
10 G-C	-0,79	-0,02	0,01	-4,74	-9,46	1,45
11 U-A	0,16	-0,19	0,38	-9,08	-5,67	-2,78
12 G-C	0,35	-0,13	0,21	4,37	-8,8	-1,19
13 C-G	0,36	-0,3	0,45	0,87	-8,54	-0,81
14 C-G	0,8	-0,47	0,43	0,38	-5,27	-0,09
Ave.	0,04	-0,19	0,20	-1,55	-11,06	-0,64

step	Shift	Slide	Rise	Tilt	Roll	Twist
1 GG/CC	-0,44	-1,71	3,29	-1,25	3,98	29,54
2 GC/GC	-0,21	-1,95	2,96	-3,28	-1,16	29,66
3 CA/UG	-0,49	-1,68	3,1	-7,28	6,21	33,58
4 AC/GU	0,28	-1,45	3,09	6,16	4,27	32,83
5 CU/AG	0,22	-1,42	3,55	-4,08	3,69	34,05
* 6 UG/GA	-2,75	-4,35	3,37	-14,72	18,48	29,64
* 7 GA/GG	0,35	0,31	6,9	-3,83	3,5	93,38
* 8 AA/UG	-1,06	-1,01	3,61	2,96	1,62	8,13
9 AG/CU	0,57	-2,02	3	-3,15	15,15	28,08
10 GU/AC	-0,6	-1,68	3,26	-1,89	6,38	32,24
11 UG/CA	0,19	-1,41	2,76	2,9	8,07	32,91
12 GC/GC	0,4	-1,65	3,19	-0,32	9,05	33,36
13 CC/GG	0,35	-1,64	3,18	1,35	6,99	31,36
Ave.	0,03	-1,66	3,14	-1,08	6,26	31,76

Supplementary Table S2: Helical parameters calculated using 3DNA, based on C1'- C1' vectors for unliganded structure ROG2.

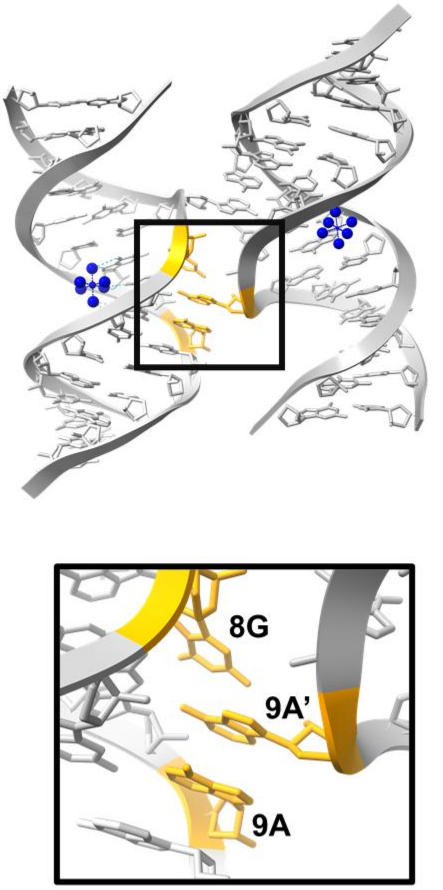

Supplementary Figure S4: Crystal lattice contacts between two neighboring RNA molecules of unliganded structure ROG2. The flipped out 9A' residue stacks between 8G and 9A residues from UGGAA motif.

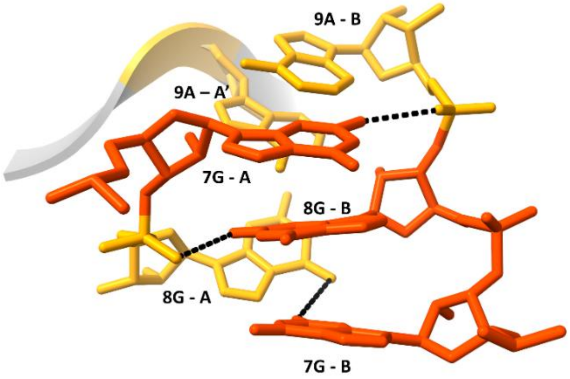

Supplementary Figure S5: Hydrogen bonds observed in cross-strand stacks in unliganded ROG2 structure

ROG 2-NCD						
bp	Shear	Stretch	Stagger	Buckle	Propeller	Opening
1 G-C	0,15	-0,13	0,46	-1,69	1,53	-2,01
2 G-C	-0,17	0,04	0,02	-1,21	-12,58	4,53
3 C-G	-0,13	-0,23	0,24	4,07	-13,51	-6,11
4 A-U	-0,35	-0,16	-0,56	-3,78	-18,63	-3,62
5 C-G	0,03	-0,04	0	-1,82	-25,78	3,12
6 U-A	0,17	0,28	0,15	-4,12	-16,42	6,19
7 A-U	0,12	-0,03	-0,04	8,5	-12,92	2,09
8 G-C	-0,41	0,03	-0,03	-3,3	-17,09	4,5
9 U-A	0,51	-0,22	0,64	-3,92	-18,25	6,09
10 G-C	0,18	0,01	0,1	-7,44	-12,65	2,34
11 C-G	0,42	-0,38	0,36	-4,26	-13,72	3,3
12 C-G	0,08	-0,02	0,16	0,89	-5,28	2,36
Ave.	0,03	-0,11	0,14	-2,25	-13,60	1,45

ROG1-NCD						
bp	Shear	Stretch	Stagger	Buckle	Propeller	Opening
1 G-C	-0,24	-0,11	0,02	-6,09	-8,78	-0,87
2 C-G	0,18	-0,16	0,08	1,58	-13,05	-1,4
3 A-U	0,04	-0,09	0,09	0,57	-12,28	1,74
4 C-G	0,26	-0,14	0,1	1,37	-18,58	1,2
5 U-A	-0,03	-0,08	0,17	-2,51	-4,25	-0,41
6 A-U	-0,31	0	0,08	0,46	-3,27	1,1
7 G-C	-0,28	-0,15	0	-0,4	-15,98	2,15
8 U-A	-0,01	-0,13	-0,01	-1,51	-13,63	5,07
9 G-C	-0,15	-0,14	-0,06	-5,64	-10,08	-0,27
10 C-G	0,05	-0,17	-0,11	1,62	-5,58	-0,66
Ave.	-0,02	-0,14	0,01	-1,06	-12,25	0,87

step	Shift	Slide	Rise	Tilt	Roll	Twist
1 GG/CC	-0,3	-1,95	3,33	2,88	4,54	29,84
2 GC/GC	-0,3	-1,44	3,2	-4,45	1,13	33,14
3 CA/UG	-0,65	-1,46	3,35	3,19	13,22	29,63
4 AC/GU	0,07	-1,34	3,25	-3,46	5,18	31,96
5 CU/AG	0,61	-1,1	3,3	1,94	7,23	34,88
6 UA/UA	----	----	----	----	----	----
7 AG/CU	-0,51	-1,31	3,57	-1,77	10,33	35,31
8 GU/AC	0,22	-1,12	3,3	-4,74	7,2	29,65
9 UG/CA	0,19	-1,35	3,12	4,47	7,6	34,63
10 GC/GC	0,5	-1,65	3,22	-0,37	1,34	30,45
11 CC/GG	-0,04	-2,2	3,03	0,98	4,06	29,61
Ave.	-0,02	-1,49	3,27	-0,13	6,18	31,91

step	Shift	Slide	Rise	Tilt	Roll	Twist
1 GC/GC	-0,74	-1,35	3,13	-1,84	0,07	33,21
2 CA/UG	0,50	-1,46	3,18	0,41	8,46	31,08
3 AC/GU	0,08	-1,36	3,24	0,99	4,19	32,37
4 CU/AG	-0,04	-1,44	3,36	-0,34	4,61	35,45
5 UA/UA	----	----	----	----	----	----
6 AG/CU	-0,44	-1,71	3,32	-1,37	5,93	31,52
7 GU/AC	-0,03	-1,31	3,29	-0,83	6,23	31,49
8 UG/CA	-0,21	-1,59	3,33	0,35	11,01	30,98
9 GC/GC	0,81	-2,31	3,06	1,86	4,70	24,22
Ave.	-0,01	-1,57	3,24	-0,10	5,65	31,29

Supplementary Table S3: Helical parameters calculated using 3DNA, based on C1’- C1’ vectors for liganded structures ROG1-NCD and ROG2-NCD.

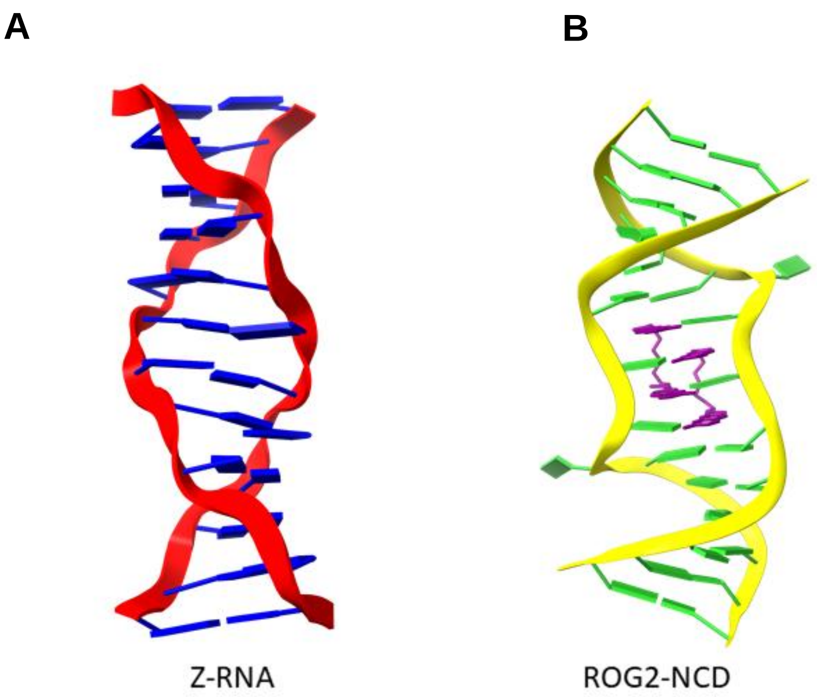

Supplementary Figure S6: Comparison of crystal structures of (A) Z-RNA form (pdb code: 4OCB) and (B) ROG2-NCD model.

Torsion angles	7G - A	8G - A	7G - B	8G - B	Z-RNA (pdb code: 4OCB)
α (O3'—P—O5'—C5')	**172.6º**	**-90.1º**	**178.5º**	**-98.6º**	**-57.3º ***
ζ (C3'—O3'—P—O5')	**-61.7º**	**-65º**	**-59.6º**	**-66º**	**-66º**

Supplementary Table S4: Torsion angles of 7G and 8G residues in liganded structure in comparison to values of torsion angles in Z-RNA structure (pdb code: 4OCB). *Torsion angle values calculated for guanosine residues in Z-RNA model.

A

	ROG2	ROG2-NCD	ROG2-$Co(NH_3)_6$
Tm **(ºC)**	79	79	79.4

B

Tm (°C)	RNA concent.	ROG2	ROG2-NCD	ROG2-$Co(NH_3)_6$	ROG1	ROG1-NCD	ROG1-$Co(NH_3)_6$
**Tm¹**	100 μM	48	-	55	33	-	39
	30 μM	41	-	46	-	-	-
	10 μM	-	62	44	-	-	-
**Tm²**	100 μM	80	77	80	65	70	67
	30 μM	80	77	79	67	65	65
	10 μM	79	81	80	69	62	68

Supplementary Table S5: Thermal stability of ROG1 and ROG2 duplexes determined by (A) Differential Scanning Calorimetry and (B) UV melting methods.

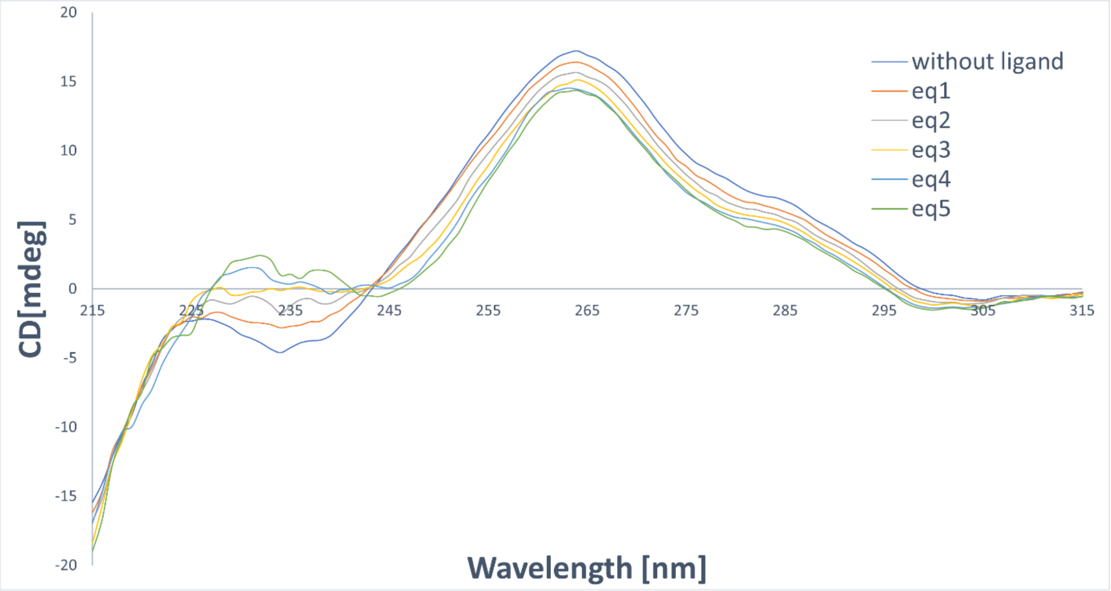

Supplementary Figure S7: Physicochemical evaluation of ROG2 duplex using circular dichroism. (D) The NCD ligand was titrated against ROG2 oligomer with molar equivalents (eq). The NCD concentration was in the range of 10-50 μM.

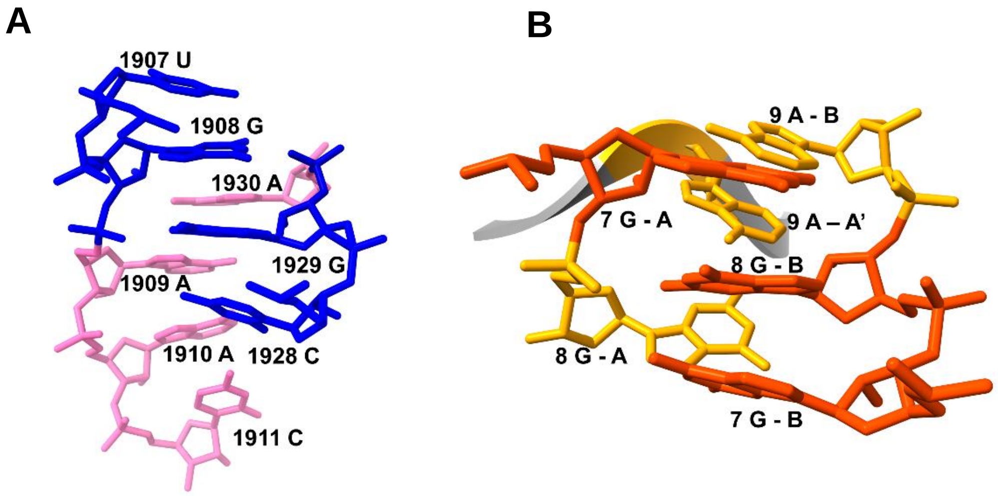

Archaebacterial rRNA from the large ribosomal subunit (LSU)

ROG2 RNA

Supplementary figure S8: Internal loops located in (A) archaebacterial rRNA from the large ribosomal subunit (LSU) and in (B) unliganded structure ROG2.
